# Evolutionary study of maize dwarf mosaic virus using nearly complete genome sequences acquired by next-generation sequencing

**DOI:** 10.1038/s41598-021-98299-9

**Published:** 2021-09-22

**Authors:** Dulanjani Wijayasekara, Akhtar Ali

**Affiliations:** 1 Department of Biological Science, The University of Tulsa, Tulsa, OK 74104 USA; 2 Department of Biological Science, The University of Tulsa, Oliphant Hall, Room N 106, 800 S Tucker Drive, Tulsa, OK 74104-3189 USA

**Keywords:** Diseases, Medical research, Urology

## Abstract

Next-generation sequencing is a robust approach to sequence plant virus genomes in a very short amount of time compared to traditional sequencing methods. Maize dwarf mosaic virus (MDMV) is one of the most important plant viruses worldwide and a significant threat to maize production. In this study, we sequenced 19 MDMV isolates (10 from Johnsongrass and 9 from maize) collected in Oklahoma and Missouri during 2017–2019 using Illumina sequencing and determined the genetic diversity. Sequence reads were assembled and 19 nearly complete genome sequences of MDMV isolates were obtained. Phylogenetic analysis based on complete genomes nucleotide and amino acid sequences revealed two main clusters and a close evolutionary relationship among 19 MDMV isolates. Statistical analysis of individual genes for site-specific selection revealed that all genes are under negative selection. The fixation index (FST) analysis of the MDMV isolates revealed no gene flow between the two main phylogenetic clusters, which emphasizes the divergence of MDMV isolates from the USA. Among the USA MDMV isolates, the mean genetic distance (d) and nucleotide diversity ((π) were highest in the P1 gene coding region. This is the first detailed study on the evolutionary relationship of MDMV isolates based on the nearly complete genome analysis from maize and Johnsongrass.

## Introduction

Next-generation sequencing (NGS) has been frequently used for plant virology studies since 2009^1–3^ and is gaining rapid recognition for discovering entire novel viral sequences and obtaining complete genome sequences for known and unknown viruses in a short period of time^4,5^. NGS provides a large number of sequences reads of virus genomes^6^, and is suitable as a novel viral detection method compared with many other methods regularly used for virus detection such as enzyme-linked immunosorbent assay (ELISA) and reverse transcription-polymerase chain reaction (RT-PCR)^4^. With regard to cost and time consumption through traditional Sanger sequencing in obtaining complete genomes of viruses, NGS is a user friendly and less time-consuming method with high resolution^4^. NGS can also be used as an easy detection method to identify low titer viruses using similarity searching against known viruses available in GenBank^7^.

Maize (*Zea mays* L.) is an important grain crop produced for several different purposes worldwide. The United States of America (USA) is the largest maize producer country in the world and maize production accounts for > 52 billion dollars with land acreage of approximately 92.0 million acres in 2020^8^. Among states, maize is cultivated on 370,000 acres in Oklahoma and 3,200,000 acres in Missouri with a production value of > 18.5 million and > 1.8 billion dollars respectively in 2019. (https://www.nass.usda.gov).

Maize infecting viruses are responsible for reducing annual maize production globally^9^. Currently, more than 50 viruses have been reported to infect maize worldwide^10^. Most of these viruses belong to the family Potyviridae*,* including maize dwarf mosaic virus (MDMV), sugarcane mosaic virus (SCMV), and wheat streak mosaic virus (WSMV)^10^. Additionally, mixed (synergistic) virus infections of maize from the same family or different virus families can have profound effects on the host. For example, Maize lethal necrosis disease is the result of a synergistic infection of maize chlorotic mottle virus (MCMV, Tombusviridae) and any of a number of members from the Potyviridae family^11,12^. Other important maize infecting plant viruses are maize chlorotic dwarf virus (MCDV, Family *Secoviridae*) and maize streak virus (MSV, Family *Geminiviridae*)^13^.

MDMV is a single-stranded, positive sense RNA genome (~ 10 kb) virus which is responsible for one of the most important viral diseases in cultivated maize and sorghum^14^ and can cause up to 70% yield loss in maize^15,16^. In 2015, MDMV resulted in the loss of 2376, 599 bushels of corn in the US and Ontario, Canada^17^. MDMV can infect more than 200 susceptible grass species including Johnsongrass (*Sorghum halepense* L. Pers) (abbreviated as JG), which is the natural overwintering host for this virus^14^.

In the USA, MDMV was first reported in 1960. Since then, it has been reported in > 37 states^18–23^. In Oklahoma, MDMV was reported for the first time from JG in 2017^24^. Subsequently, we collected a large number of MDMV isolates from both maize and JG and determined the genetic diversity of MDMV isolates based on the coat protein (CP) gene sequences^25^. We also reported the first MDMV complete genome from JG and together there are only seven complete genomes of MDMV available in GenBank, where only one of them is from JG, while all others are from maize^25^. There is no comprehensive study revealing evolutionary relationships of these MDMV isolates due to a lack of additional complete genome sequences. It is important to study the complete genome sequences of MDMV isolates in both maize and JG to understand if the virus is mutating and moving towards being novel strains.

Therefore, in this study, we sequenced 19 nearly complete MDMV genomes from both maize and JG using Illumina sequencing, which contained the full coding region of the virus with varying 5′ and 3′ untranslated regions. Phylogenetic relationships and selection pressure based on coding sequences of the genomes as well as on individual genes sequences of MDMV isolates were analyzed.

## Results

### Field symptoms of MDMV

**Figure 1:**
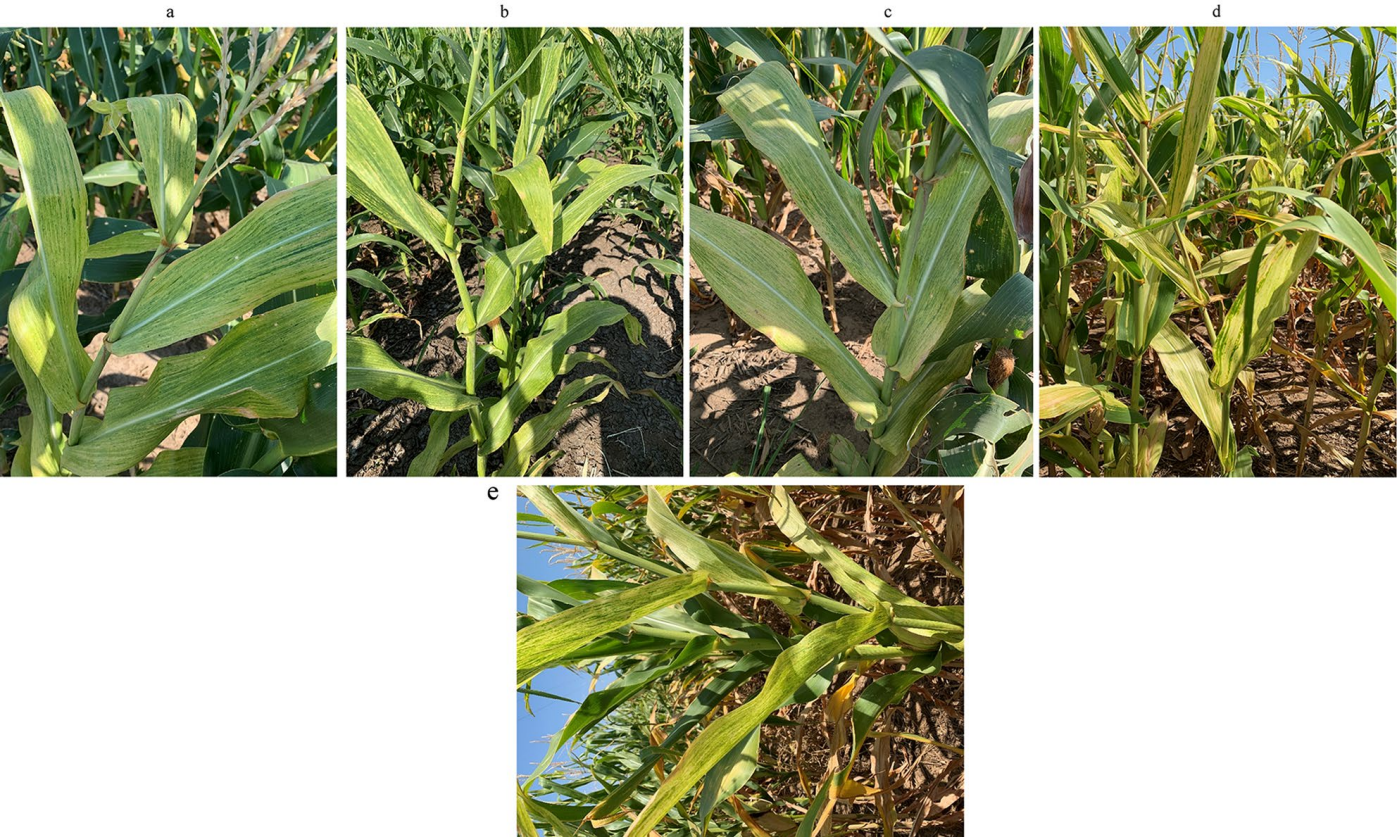
Field symptoms of MDMV-infected maize samples (**a–e**) collected during 2019 growing season from different fields in Kay County in Oklahoma showing typical yellow streaking, chlorosis and mild mosaic on leaf blades.

The most common field symptoms of MDMV in both hosts (JG and maize) were yellow streaks, chlorosis, and mild to severe mosaic on the leaf blades. Symptoms of a few samples from the maize plants, which were collected in different locations in Oklahoma, are shown in Fig. 1a–e, Symptoms of MDMV on JG have been reported in our previous work^25^.

### VLPs and virion RNA isolation

**Figure 2:**
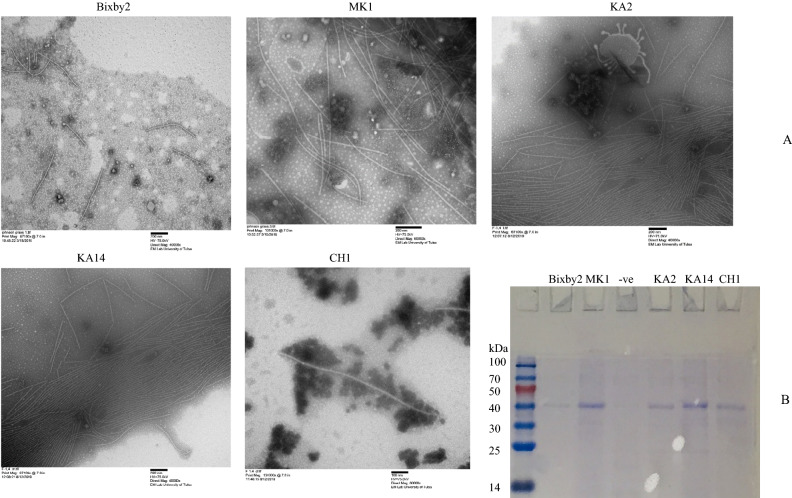
(**A**) Analysis of virus-like particles (VLPs) preparations by transmission electron microscope isolated from infected Johnsongrass and maize showing flexuous filamentous virus particles. (a) MDMV Bixby-2 isolate from Johnsongrass collected in Tulsa County in 2018, (b) MDMV MK1 isolate from Johnsongrass collected in Muskogee County in 2018, (c,d) MDMV KA14 and KA2 isolates from maize collected in Kay County in 2019, (e) MDMV-CH1 isolate from maize collected in Cherokee County in 2018 (Details of MDMV isolates-Table 1). (**B**) Analysis of virus-like particles (VLPs) on 12% SDS-PAGE stained with Coomassie Brilliant Blue. All VLPs samples showed a band of capsid protein (≈ 39 kDa) from MDMV isolates from Johnsongrass and maize collected in various counties in Oklahoma. Lane 1: Pre-stained SDSPAGE protein standards (GenScript, NJ, USA). Lane 2: MDMV-Bibxy2 isolate from Johnsongrass, Lane 3: MDMV MK1 isolate from Johnsongrass, Lane 3: No band from healthy maize used as a negative control. Lanes 4 and 5: MDMV KA14 and KA2 isolates from maize respectively, and Lane 6: MDMV CH1 isolate from maize (Details of MDMV isolates-Table 1).

Virus-like particles (VLPs) preparations were successfully obtained from all 19 selected dot-immunobinding assay (DIBA) positive MDMV samples. VLPs from a few MDMV isolates were observed by electron microscope and all of them contained typical long flexuous filamentous particles of MDMV (Fig. 2A). VLPs from all MDMV isolates (Table 1) were run on an SDS-PAGE gel and showed the presence of probable capsid protein for MDMV. VLPs of selected MDMV isolates showing capsid protein of approximately 39 kDa has been shown in Fig. 2B. RNA was successfully extracted from all 19 VLPs. The quality of viral RNA was in the range of 1.9–2.0 at 260/280 ratio and the concentration ranged from 10 to 50 ng/µl.

**Table 1:** Complete genome sequences of Maize dwarf mosaic virus (MDMV) isolates.

Country	State	County	Isolate	Year collected	Host	Total (nucleotides)	39 insertion	Accession no.	References
Bulgaria	–	–	Bulgaria	–	Corn	9515		NC_003377	^28^
Hungary	–	–	Sz0605	2006	Corn	9515		FM883211	^26^
Hungary	–	–	Mv0801	2008	Corn	9554	Present	FM883164	^26^
Iran	–	–	Golestan	2010	Corn	9499		JQ280313	^49^
Italy	–	–	Italy	2009	Corn	9491		JX185302	^36^
Spain	–	–	SP	1992	Corn	9523		AM110758	^50^
USA	Ohio	–	OH-1	1960	Corn	9458		JQ403608	^36^
USA	Ohio	–	OH-2	1960	Corn	9462		JQ403609	^36^
USA	Oklahoma	Tulsa	Bixby1	2018	Johnsongrass	9563	Present	MK282423	^25^
USA	Oklahoma	Tulsa	Bixby2	2018	Johnsongrass	9225	Present	MW026050	This study
USA	Oklahoma	Tulsa	Bixby3	2018	Johnsongrass	9497	Present	MW026051	This study
USA	Oklahoma	Cherokee	CH1	2019	Corn	9453	Present	MW026060	This study
USA	Oklahoma	Kay	KA1	2019	Corn	9454	Present	MW026061	This study
USA	Oklahoma	Kay	KA2	2019	Corn	9391	Present	MW026062	This study
USA	Oklahoma	Kay	KA11	2019	Corn	9372	Present	MW026063	This study
USA	Oklahoma	Kay	KA12	2019	Corn	9431	Present	MW026064	This study
USA	Oklahoma	Kay	KA13	2019	Corn	9395	Present	MW026065	This study
USA	Oklahoma	Kay	KA14	2019	Corn	9440	Present	MW026066	This study
USA	Oklahoma	Kay	KA15	2019	Corn	9393	Present	MW026067	This study
USA	Oklahoma	Kay	KA16	2019	Corn	9439	Present	MW026068	This study
USA	Oklahoma	Muskogee	MK1	2018	Johnsongrass	9394	Present	MW026054	This study
USA	Oklahoma	Osage	OS1	2018	Johnsongrass	9415	Present	MW026055	This study
USA	Oklahoma	Payne	PY1	2018	Johnsongrass	9413	Present	MW026056	This study
USA	Oklahoma	Payne	PY2	2018	Johnsongrass	9205	Present	MW026057	This study
USA	Oklahoma	Payne	PY3	2018	Johnsongrass	9430	Present	MW026058	This study
USA	Oklahoma	Payne	PY4	2018	Johnsongrass	9444	Present	MW026059	This study
USA	Missouri	McDonald	MD1	2018	Johnsongrass	9450	Present	MW026052	This study
USA	Missouri	McDonald	MD2	2018	Johnsongrass	9491	Present	MW026053	This study

### Next-generation sequencing and data assembly

Next generation sequencing produced a nearly complete genome sequence for the 19 MDMV isolates from both maize and JG using 19 different Illumina libraries (Supplementary Table S1, Table 1). Assembled complete genomes were BLASTed against NCBI database and percent nucleotide (nt) identity to NCBI reported MDMV isolate Bixby1 (MK282423) varied from 94 to 96%, while none of the contigs showed homology to other maize viruses indicating that these samples are only infected with MDMV (Supplementary Table S1). The total number of 160,690–1,533,638 reads and 23,490,807–221,249,681 bases were obtained for MDMV isolates from maize. Additionally, the total number of 196,152–1,303,612 reads and 28,328,952–188,119,485 bases for MDMV isolates were from JG. Computed % coverage of viral contigs from both maize and JG isolates ranged from 4–37% to 6–35% of the total sequence was MDMV, respectively.

### Nearly complete genomes of MDMV isolates

The length of the genomes among 19 MDMV isolates varied from 9204 to 9497 nucleotides (nt) (Table 1). In all MDMV contigs, the length of ORFs were the same, but differences were noticed in the lengths of the 5′ and 3′ untranslated regions. The variability in the UTRs may not be an accurate representation of these genomes because no 5′ and 3′ RACE-PCR was conducted and is probably the result of differences in the assemblies Intact coding regions of polyprotein for these 19 genomes showed 94–95% identity to the MDMV-Bixby1 isolate (accession no. MK282423). All MDMV sequences obtained in this study were deposited in GenBank with the accession numbers MW026050–MW026068 (Supplementary Table S1). All USA MDMV isolates contained an additional 39 nt in the N terminal region of the CP gene as described before^25,26^. The length of the polyprotein for all 19 isolates was 3053 amino acids (aa), which is cleaved into ten different protein products including P1, P3, 6K1, 6K2, CI, CP, HC-Pro, NIA Pro, NIa VPg, NIb and PIPO as reported before^27,28^.

### Phylogenetic analysis of MDMV isolates

**Figure 3:**
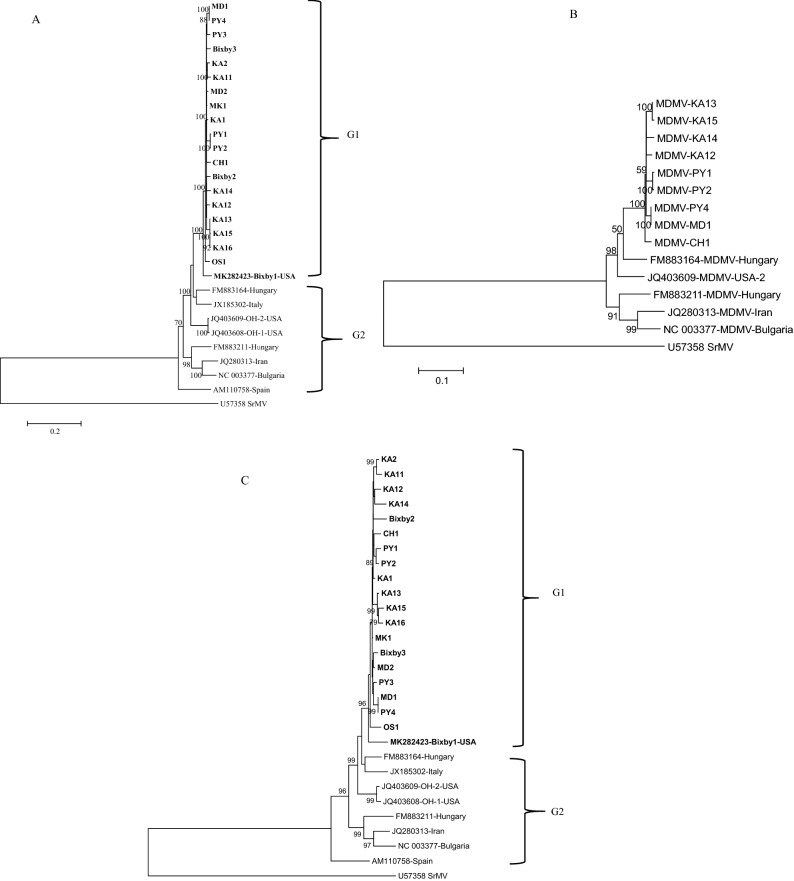
Maximum likelihood (ML) trees based on complete open reading frame nucleotide sequences with (**A**) all 19 MDMV recombinant isolates, (**B**) without out recombinant MDMV isolates (**C**) and amino acids sequences depicting evolutionary relationships between the 19 maize dwarf mosaic virus (MDMV) isolates from maize and Johnsongrass sequenced in this study and nine MDMV isolates (retrieved from GenBank) (Accession and abbreviation are listed in Table 1). The tree was constructed in MEGA6 program using Tamura Nei model with 1000 bootstrap replications. The GenBank accession no. of each isolate is followed by name of the country.

Both neighbor joining (NJ) and maximum likelihood (ML) trees were developed for the nt sequences of the entire ORF with a total of 28 MDMV isolates (19 in this study and 9 retrieved from GenBank) plus Sorghum mosaic virus (SrMV) as an out-group. The topology of the NJ and ML trees were almost identical with robust bootstrap support values. The ML phylogenetic trees based on the complete nt polyprotein sequences with all the isolates (Fig. 3A) or without recombinant isolates (Fig. 3B) as well as amino acid sequences (Fig. 3C) revealed two main phylogroups named G1 and G2. Phylogroup G1 contains all 20 MDMV isolates from maize and JG collected in Oklahoma and Missouri, USA (19 in this study) along with one MDMV isolate from our previous study collected from JG in 2018 (MDMV Bixby1 isolate, accession no. MK282423).

Phylogroup G2 contained the eight remaining MDMV isolates, including two each from the USA and Hungary, and one each from Italy, Iran, Bulgaria, and Spain (Fig. 3A). The genetic distance (d) value for the entire G1 group including 20 MDMV isolates was 0.027 ± 0.001, while for the G2 group, the d value was 0.142 ± 0.003. Members of the G2 group have diverged more from each other compared to members of the G1 group, which has a lower d value indicating closer relatedness to each other. A large number of coding regions of both phylogroups were under negative selection, while the dN/dS ratios for individual phylogroups were below 1, which also indicates that these isolates are under negative selection. The fixation index (*FST)* value between G1 and G2 phylogroups was 0.358, indicating no apparent gene flow (Table 2), and was above the threshold of *FST* > 0.33 value^29,30^.

**Table 2:** Selection pressure and genetic distance and diversity of two main phylogroups of MDMV.

Popul ation	dN^c^	dS^d^	dN/dS^e^	FST	Number of negatively selected codons^f^				Number of positively selected codons^g^				Nucleoti de diversity (π)^h^	Mean value for genetic distance (d)
					SLAC	MEME	BUSTED	FEL	SLAC	MEME	BUSTED	FEL		
G1^a^	0.0476	0.9941	0.0478	0.358	188	0	0	572	0	27	1	4	0.02539	0.027 ± 0.001
G2^b^	0.1884	2.3466	0.0802		584	0	0	1534	0	99	1	4	0.11697	0.142 ± 0.003

^a^G1 20 MDMV isolates bG2 8 MDMV isolates.

^c,d,e^dN, dS and the dN/dS ratio were determined by DnaSP6.

^f,g^Number of negatively and positively selected codons, obtained using the SLAC, MEME, BUSTED and FEL methods implemented in Data monkey.

^h^According to Nei^44^.

**Figure 4:**
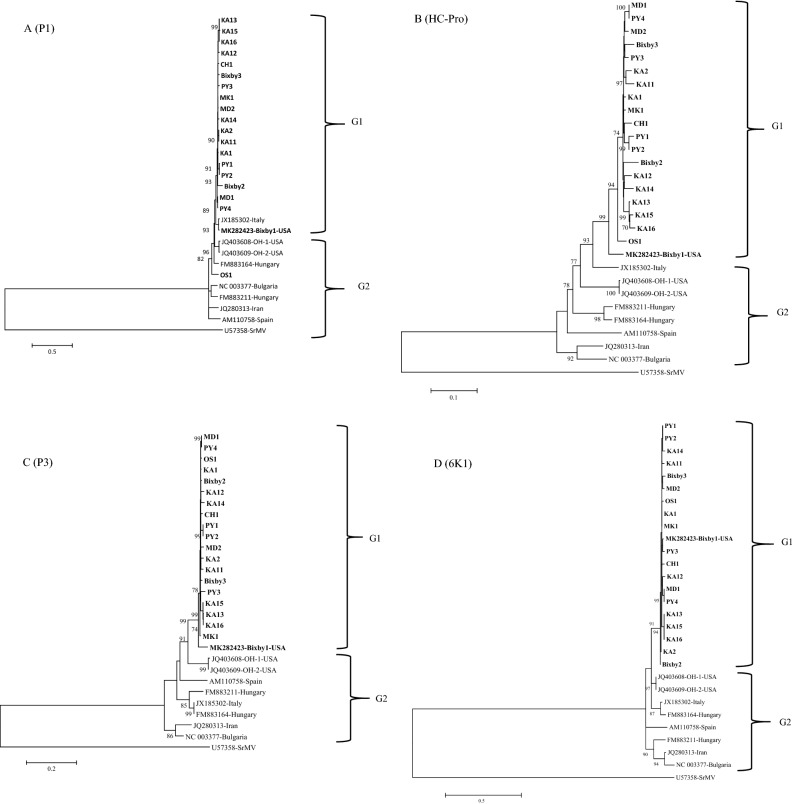

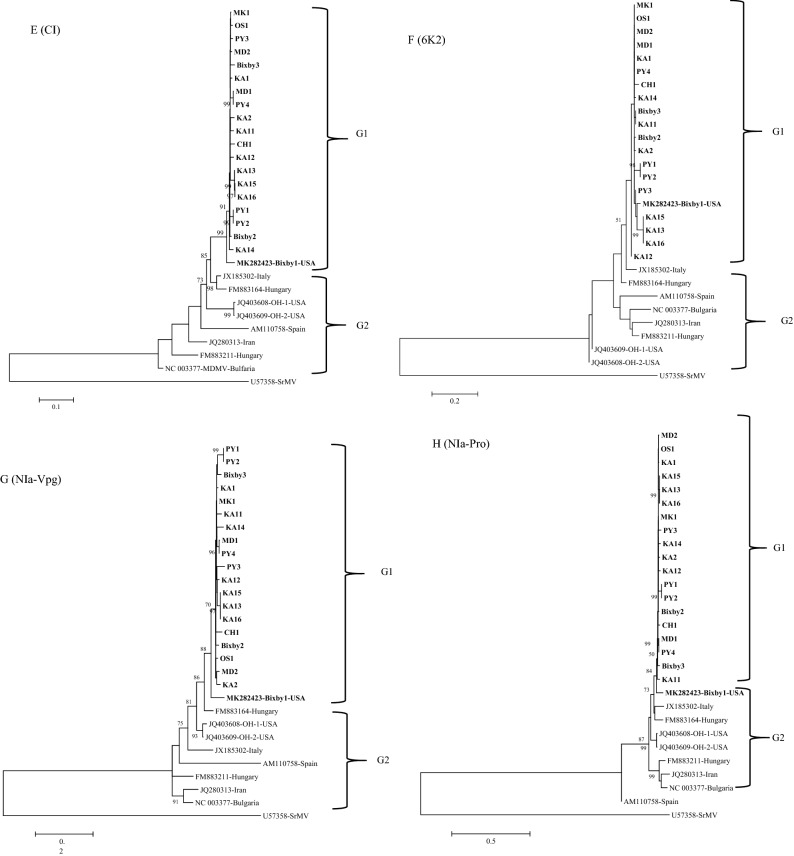

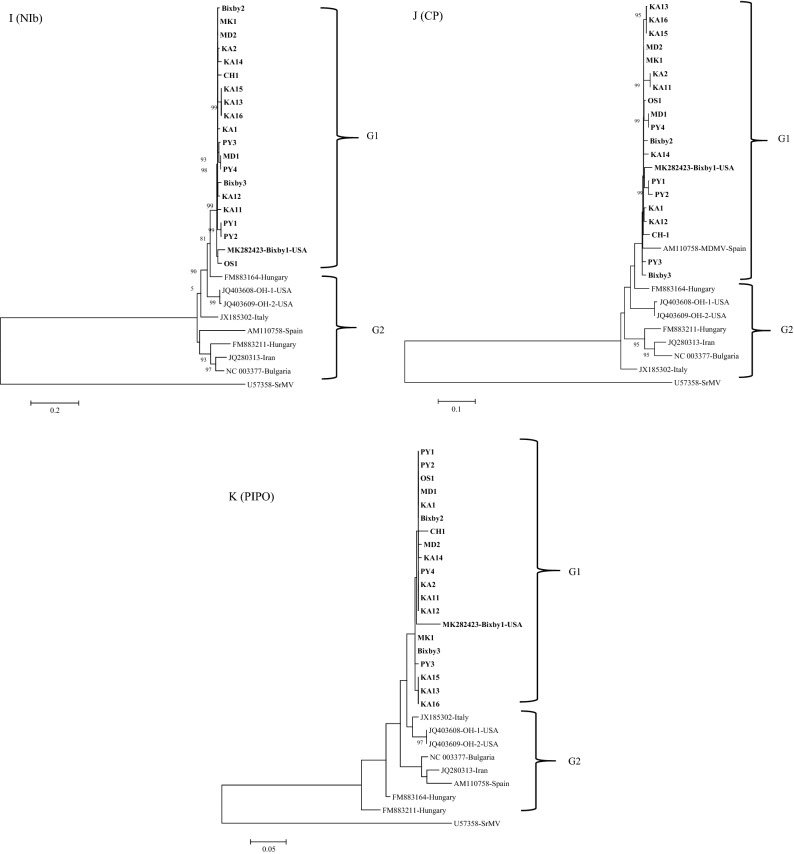
Maximum likelihood (ML) trees based on the nucleotide sequences of individual genes of 19 maize dwarf mosaic virus (MDMV) from maize and Johnsongrass sequenced in this study and nine MDMV isolates (downloaded from GenBank): (**A**) P1, (**B**) P3, (**C**) HC-Pro, (**D**) CI, (**E**) CP, (**F**) NIb, (**G**) NIa-Pro, (**H**) NIa-Vpg, (**I**) 6K1, (**J**) 6K2, (**K**) PIPO. The tree was constructed in MEGA6 program using Tamura Nei model with 1000 bootstrap replications. The GenBank accession no. of each isolate is followed by name of the country.

Within G1, three MDMV isolates KA13, KA15 and KA16 (Fig. 3A–C) clustered together similar to individual gene trees (Fig. 4A–K). These three isolates were collected from the same Kay County of Oklahoma (Table 1) in two different fields, where KA15 and KA16 were collected from the same field, and KA13 was collected from a different field. MDMV isolates in the G1 group do not indicate clear segregation based on the host or the collection year. Isolates collected from both hosts (maize and JG) in different years clustered together in the G1 phylogroup.

Individual gene phylogenetic trees of 28 MDMV isolates showed (Fig. 4A–K) a similar pattern to the complete genome tree except for the P1 and CP genes trees. In the P1 gene tree, the MDMV OS1 isolate clustered in G2, while the MDMV isolate from Italy clustered in the G1 phylogroup. Similarly, in the CP gene tree an MDMV isolate from Spain clustered in the G1 phylogroup. Within G1, variation in branching pattern can be observed based on individual gene trees. For example, KA13, KA15, and KA16 MDMV isolates consistently grouped into one sub-cluster within the G1 phylogroup across all individual gene trees (Fig. 4A–K). There was no clear clustering of MDMV isolates based on their host, but this could be on the basis of location as evident from phylogenetic trees (Figs. 3A–C, 4A–K, Supplementary Fig. S1).

### Gene by gene selection analysis

In order to evaluate selection pressure on each coding region for the individual gene as well as the entire coding region of 28 MDMV isolates used in this study, the dN/dS ratios and number of negative and positive selected codons were identified using several analytical methods using the Datamonkey 2.0 online server (Table 3). The dN/dS ratio for all individual genes indicated high negative selections among the MDMV isolates. The P1 region was found to have the highest dN/dS value (0.1829), while the lowest was found for the CI coding region (0.0089). Four different analytical methods, including BUSTED, MEME, SLAC and FEL, were used to identify positively and negatively selected codons. Both SLAC and FEL identified a large number of negatively selected codons. All of these methods indicate highly purifying selection governing in all individual genes of MDMV. Both MEME and BUSTED identified a larger number of codons under neutral selection with a fewer number of codons under positive selection for regions of HC-Pro, P3 and CP.

**Table 3:** Selection pressure analysis for each coding region of 28 MDMV complete genomes sequences.

Coding region	ENC	dN^a^	dS^a^	dN/dS^a^	Number of negatively selected codons^b^				Number of positively selected codons^b^			
					SLAC	MEME	BUSTED	FEL	SLAC	MEME	BUSTED	FEL
P1	233	0.0487	0.2662	0.1829	68	0	0	90	0	1	0	1
HC-Pro	460	0.0138	0.5985	0.0230	171	0	0	284	1	3	1	2
P3	347	0.0181	0.3761	0.0481	70	0	0	155	0	1	1	1
6K1	67	0.0139	0.9069	0.0153	18	0	0	40	0	0	0	0
CI	638	0.0044	0.4904	0.0089	244	0	0	427	0	2	0	0
6K2	53	0.0209	0.5952	0.0351	9	0	0	27	0	0	0	0
NIa-VPg	189	0.0202	0.5504	0.0367	58	0	0	107	0	0	0	0
NIa-Pro	242	0.0086	0.4411	0.0194	65	0	0	123	0	1	0	0
NIb	521	0.0117	0.4601	0.0254	154	0	0	288	0	0	0	0
CP	304	0.0096	0.2740	0.0350	61	0	0	122	0	2	1	1
PIPO	79	0.0292	0.1093	0.2671	3	0	0	9	0	1	1	0

*ENC* effective number of codons.

^a^dN, dS and the dN/dS ratio were determined by DnaSP6.

^b^Number of negatively and positively selected codons, obtained using the SLAC, MEME, BUSTED and FEL methods implemented in Data monkey.

Further analysis was carried out for selected populations of MDMV to understand nucleotide diversity (π) and genetic distances (d) in individual genes (Table 4). Among the US isolates (20 MDMV isolates), the P1 coding region had the highest nucleotide diversity and genetic distance compared to other individual genes. For the MDMV population from other countries, 6K2 had the highest nucleotide diversity (0.13612), while P1 had the highest genetic distance (0.166 ± 0.012). The MDMV population based on their hosts (20 US MDMV isolates) indicated that HC-Pro had the highest nucleotide diversity and genetic distance in maize MDMV population, while for JG population it was the P1 coding region.

**Table 4:** Population based analysis of genetic parameters for individual coding region of 28 MDMV isolates.

Gene	US^b^ isolates	Nucleotide diversity (π)^a^			All together	US isolates	Overall mean Genetic distance (d)			All together
		International^c^ isolates	Maize^d^ isolates	JG^e^ isolates			International isolates	Maize isolates	JG isolates	
P1	0.04011	0.13085	0.02265	0.05111	0.07922	0.045 ± 0.004	0.166 ± 0.012	0.024 ± 0.003	0.059 ± 0.005	0.097 ± 0.007
HC-Pro	0.03528	0.12549	0.03142	0.03655	0.08110	0.038 ± 0.002	0.055 ± 0.009	0.033 ± 0.003	0.039 ± 0.003	0.096 ± 0.005
P3	0.01959	0.11287	0.01831	0.01988	0.06714	0.021 ± 0.002	0.135 ± 0.009	0.019 ± 0.003	0.021 ± 0.002	0.078 ± 0.005
6K1	0.02252	0.12331	0.02349	0.02026	0.07415	0.024 ± 0.005	0.140 ± 0.022	0.025 ± 0.007	0.021 ± 0.005	0.079 ± 0.014
CI	0.02095	0.11425	0.02157	0.01889	0.06584	0.022 ± 0.002	0.130 ± 0.007	0.023 ± 0.002	0.015 ± 0.002	0.073 ± 0.044
6K2	0.02297	0.13612	0.03005	0.01601	0.07201	0.025 ± 0.006	0.075 ± 0.029	0.033 ± 0.010	0.017 ± 0.006	0.085 ± 0.014
NIa-VPg	0.03013	0.12623	0.02562	0.03312	0.07542	0.032 ± 0.003	0.164 ± 0.013	0.027 ± 0.004	0.036 ± 0.004	0.094 ± 0.007
NIa-Pro	0.02205	0.10921	0.01779	0.02424	0.06124	0.023 ± 0.003	0.138 ± 0.011	0.018 ± 0.003	0.026 ± 0.003	0.074 ± 0.006
NIb	0.02452	0.12090	0.02243	0.02448	0.06795	0.026 ± 0.002	0.155 ± 0.013	0.023 ± 0.003	0.026 ± 0.002	0.083 ± 0.007
CP	0.01968	0.08188	0.01837	0.01950	0.04594	0.020 ± 0.002	0.095 ± 0.009	0.019 ± 0.003	0.020 ± 0.003	0.051 ± 0.005
PIPO	0.00906	0.05937	0.00762	0.00982	0.03013	0.009 ± 0.003	0.062 ± 0.012	0.008 ± 0.004	0.010 ± 0.004	0.031 ± 0.006
Complete ORF	0.02562	0.11697	0.02273	0.02642	0.06842	0.027 ± 0.001	0.142 ± 0.003	0.024 ± 0.001	0.028 ± 0.001	0.080 ± 0.002

^a^According to Nei^44^.

^b^20 MDMV isolates obtained from Maize and Johnsongrass (Fig. 1, Table 1).

^c^Eight MDMV isolates used for comparison (Fig. 1, Table 1).

^d^Eight MDMV isolates obtained from Maize in this study (Fig. 1, Table 1).

^e^12 MDMV isolates obtained from Johnsongrass (11 in this study and one in previous study) (Fig. 1a, Table 1).

### Analysis of recombination among MDMV isolates

A total of 36 recombination events were detected when 28 complete genomes sequences of MDMV were analyzed by RDP4.69 software (Table 5). Among those recombination events, only 10 were found to be significant because they were detected by at least five or more RDP implemented programs with significant recombination events (Table 5). Most of the recombination events were located in the P1 and HC-Pro genes and some were in the CP and 3’UTR regions involving mostly the USA MDMV isolates.

**Table 5:** Putative recombination events among 28 complete genomes of MDMV isolates detected by RDP4.69 software.

Event #	Putative recombinant	Putative parents		Detected by	P-value
		Major	Minor		
1	MW026055-OS1	MW026061-KA1	JQ403608-OH-1	R–B M C S T	1.614 × 10^–18^
2	AM110758-Spain	JX185302-Italy	MW026054-MK1	R G B M C S T	1.787 × 10^–18^
3	MW026063-KA11	MW026054-MK1	MW026062-KA2	R G B M C S T	1.352 × 10^–16^
4	MW026050-Bixby2	MW026054-MK1	MK282423-Bixby1	R G B M C S T	1.981 × 10^–16^
5	MW026055-OS1	MW026054-MK1	FM883211-Hungary	R G B M C–T	1.507 × 10^–15^
6	JX185302-Italy	MW026059-PY4	MK282423-Bixby1	R G–M C–T	3.753 × 10^–14^
7	MW026050-Bixby2	MW026068-KA16	Mw026051-Bixby3	R G B M C–T	9.418 × 10^–8^
8	JX185302-Italy	JQ403608-OH-1	MW026058-PY3	R–B M C S T	2.064 × 10^–7^
9	MW026051-Bixby3	MW026053-MD2	MK282423-Bixby1	R G B M C–T	6.889 × 10^–5^
10	MW026054-MK1	MW026061-KA1	MW26053-MD2	–G–M C S T	2.994 × 10^–6^

Only ten recombination events that involved 19 MDMV isolates in this study are shown in this table, which were detected by at least five of the seven suits implemented in the RDP4.69 with a good statistical support (*P* ≤ 0.05).

## Discussion

Within the span of 40 years, DNA sequencing has improved dramatically. NGS, is more expensive compared to traditional Sanger sequencing, but efficiently provides a large amount of data. Earlier next generation sequencing methods such as 454 pyrosequencing were used to quickly cover entire viral genomes, which helped dramatically in novel virus discovery, plant virus biodiversity, and evolutionary and ecology-based studies^6^. Currently, many novel plant virus discoveries have successfully and efficiently used Illumina sequencing^7,31^. Therefore, the 19 MDMV genomes sequenced in this study using Illumina sequencing provided sequences in performing evolutionary studies on such a range of MDMV isolates.

MDMV is the most common among virus diseases of maize and can infect more than 200 grass species^14,26,32^. Complete genome sequence information is important to understand the virus evolution based on the entire genome as well as individual genes of a virus and their molecular relationships with other available isolates collected from different regions and countries. Therefore, in this work, an additional 19 MDMV isolates were sequenced and determined their relationships with the available MDMV isolates in the GenBank. The complete ORF of 28 MDMV isolates has indicated strong evolutionary relationship between isolates that contained the 39 nt insertion in the N terminal region of the CP gene (G1 phylogroup) and on the origin from where they were collected as reported in our previous study^25^. Within the G1 phylogroup, MDMV isolates from both maize and JG clustered together showing no host preferences as observed previously in the Hungarian MDMV isolates^26^. These results indicate that MDMV isolates may frequently originating from JG and moving towards maize by vector transmission but further experimental evidence is needed in future studies.

In G2 phylogroups, MDMV isolates were polyphyletic but more complete genome sequences of MDMV isolates from these countries are needed to get a better understanding of phylogenetic relationships within the G2 phylogroup. Similar evolutionary relationships were obtained based on the CP gene, where MDMV isolates clustered into two main groups based on the 39 nt insertion in the CP gene^25^.

An individual phylogenetic tree for each gene of the MDMV isolates showed a similar tree topology with the one obtained from complete genome sequences (Fig. 3A–C). Further analysis of individual genes for their genetic diversity (π), mean genetic distance (d), and selection pressure in different MDMV populations (Table 5) indicated the P1 gene is associated with the greatest genetic diversity among US isolates and therefore, is the most phylogenetically informative. All the individual genes were under negative selection, where NIb, HC- Pro and CI have the highest number of negatively selected codons. These genes are associated with viral genome replication and are under strong purifying selection to assure viral genome replication. P1 and CP genes have less negatively selected codons compared to other genes, indicating these genes might allow some variation within the coding region and could be more informative in an evolutionary prospective.

Analysis of a larger number of complete genome sequences has improved the resolution of evolutionary relationships among MDMV isolates, where isolates are grouped into G1 and G2 phylogroups (Fig. 3A,B). The tree topology of the G1 phylogroup did not vary with or without recombinant MDMV isolates obtained in this study (Fig. 3A,B), indicating that the USA MDMV isolates are unique from MDMV isolates reported from other countries. These results were further supported by ML tree obtained from complete aa sequences of MDMV isolates (Fig. 3C). Recombination analysis of all MDMV isolates showed that significant recombination events were observed among USA MDMV isolates (Table 5). This means that recombination could be a major reason for greater diversity observed among USA MDMV isolates. In addition to mutations, recombination could be a major factor contributing to the emergence of a unique MDMV population in the USA, as is evident in Fig. 3A–C where USA isolates cluster separately from the rest of the MDMV isolates reported from other countries.

The use of the CP gene for the generation of MDMV phylogenies, may be appropriate in cases where complete genomes are not available, due to the congruency observed between the CP and complete genome trees from this study. The CP gene of plant viruses has been used in many evolutionary studies in the past because the N terminal region is very informative with high genomic variability^14,26,33–35^, while the core region is more stable, providing enough information in understanding MDMV evolution^25^. In the case of MDMV, the CP gene is evolutionary more informative with the 39 nt insertion as shown in Supplementary Fig. S1, which is a clear evolutionary event where all the isolates that do not have the insertion are ancestral in origin^25^. All the USA MDMV isolates, whether sequenced previously^25^ or in this study, were collected from Oklahoma and Missouri and contained the 39 nt insertion in the CP gene, which is known to be a duplication event that happened at one point in the virus evolution^26,35^, and suggests that this insertion stabilized over time. The length of the CP gene can vary due to the presence of this 39 nt insertion (i.e. < 876, 888 and 915 nt; 292, 296 and 305 aa, respectively)^26,35^. For example, MDMV OH-1 and 2 isolates (Table 1) had shorter CP gene sequences lacking 39 nt insertion and both of these isolates were maintained using successive mechanical inoculations^36^. MDMV survives in JG rhizomes during the winter, and in the spring, aphids transmit the virus in a non-persistent manner to maize. MDMV isolates from two states in this study contained 39 nt insertion in the CP gene and caused severe maize dwarf disease in susceptible maize cultivars. We previously proved the aphid transmissibility of the MDMV Bixby1 isolate, which has the 39 nt insertion^25^.

In conclusion, this is the first study that uses a larger number of MDMV isolates from both maize and Johnsongrass and has provided a detailed evolutionary analysis of the nearly complete genome sequences as well as of individual genes of the virus. Our study has shown a better understanding of the molecular diversities and population structures among MDMV isolates as well as the best candidate gene for MDMV phylogeny for future studies. In addition, this work confirms the stability of a 39 nt insertion in the N terminal region of the CP and its importance in MDMV evolution. However, further studies could focus on understanding the function of the 39 nt insertion in the CP gene for better understanding of its contribution in MDMV evolution and severity of maize dwarf disease. The information obtained in this work would be helpful in the management strategies of MDMV in maize, and for future evolutionary studies of MDMV isolates reported worldwide.

## Materials and methods

### Sample collection

Maize and JG leaf tissues were collected from symptomatic plants in six different counties within Oklahoma and one County in Missouri. A total of 106 samples were collected from JG in 2018 and 45 samples from maize in 2019 as reported in our previous work^25^. Tissue samples were brought back to the lab on ice and stored in − 20 ℃ freezer for further usage.

### Serological detection

All of the collected maize and JG samples were tested by DIBA as described in our previous study^25,37^. A total of 19 MDMV DIBA positive samples (Table 1) were randomly selected as a representative sample from each of the seven counties in two states based on the number of fields, location and states and used it for further molecular characterization in this study (Table 1).

### Virus like particle preparation, SDS-PAGE and virion RNA extraction

One to two grams tissues from each of the 19 MDMV DIBA positive samples were used for virus like particles (VLPs) preparation as described before^38^. VLPs pellets from each individual MDMV isolate was resuspended in 50–200 µl of nuclease free water. To observe virion particle morphology, few VLPs samples were checked on Transmission Electron Microscope (TEM) according to procedure reported previously^39^. To confirm the presence of capsid protein, an aliquot of 10 µl of each VLPs suspension was mixed with equal volume of 2 × SDS loading dye^40^, run on 12% SDS-PAGE and stained as described before^39^.

After confirming the presence of capsid protein on SDS-PAGE, purified MDMV RNA was extracted from VLPs of each MDMV isolate using RNeasy Plant Mini Kit (Qiagen, MD, USA), and according to the manufacture’s protocol, 100 µl of VLPs was mixed with 450 µl of RLT buffer. Viral RNA was eluted in 30 µl of RNase free water. The quality of RNA (using 260/280 ratio) and concentration was determined using NanoDrop8000 Spectrophotometer (Thermo Fischer Scientific, Waltham, MA, USA).

### Illumina Nextera XT library preparation

Two step reverse transcription-polymerase chain reaction (RT-PCR) was performed with random hexamers to cover the complete genome of MDMV. The RT step including 10 µl of viral RNA, 4 µl 5× RT buffer, 2 µl of 10 µM dNTPs; 2.0 µl of 20 µM of random-hexamers primer, 1.0 µl of RNase inhibitors and 1.0 µl MMLV reverse transcriptase according to the manufacturer’s instructions (Genscript, Piscataway, NJ, USA). RT-PCR was carried out in a 20 µl reaction using 1 U of Taq DNA polymerase (Genscript, Piscataway NJ, USA), 1× Taq buffer, 0.2 mM dNTPs, 5.0 µM random-hexamer primer, and 2 µl of template cDNA. Thermal cycling conditions were 35 cycles of 95 °C for 30 s, annealing 45 °C for 30 s, and extension 72 °C 30 s. The PCR products were analyzed on 1% agarose gel and smears of DNA (ranges from 300 to 1500 bp) were purified using gel extraction kit (Promega, USA).

Purified DNA was quantified using Qubit dsDNA HS Assay Kit (Thermo Fisher Scientific, Waltham, MA, USA). A total of 19 individual DNA libraries for each of the samples were prepared using Nextera XT DNA Library Preparation Kit (Illumina, CA, USA). Individual samples were dual-indexed with unique barcodes for identification purposes of recovered complete genome from each individual sample. Amplified and index adapted libraries were purified using AMpure XP beads and quantified using Qubit dsDNA HS Assay Kit (Thermo Fisher Scientific, Waltham, MA. USA). Individual libraries were pooled and sequenced using pair-end sequencing (2 × 150 bp) using single flow cell on the Illumina MiSeq (San Diego, CA, USA).

### Assembly and mapping of Illumina sequencing reads

Raw reads from Illumina sequencing were trimmed and assembled using CLC Genomics Workbench (CLCGW v12.0.3) (Qiagen, MD, US). Both forward and reverse sequences were paired with the following parameters: paired end (forward-reverse) minimal distance 1 to maximum distance 1000, while quality scores were kept at Illumina Pipeline 1.8 and later. Paired reads were assembled using the following parameters: reads were assembled using the de novo assembly function on CLCGW with default graph parameters (automatic word size, and automatic bubble size parameters), and for paired reads, auto-detect paired distances and perform scaffolding functions were selected. Minimum contig’s length was set to 200, mismatch cost two, insertion cost three, deletion cost three, length fraction 0.5, and similarity fraction 0.99. Reference-based mapping was then carried out using complete genome sequences available on GenBank for several maize infecting viruses including MDMV, MCMV, SCMV and WSMV (MK282423, JX286709, KP860936, NC_001886). Mapping parameters were set as follows: for read alignment, match score was set at one while mismatch cost two with linear gap cost function selected with an insertion cost of three, deletion cost of three, length fraction at 0.5, and similarity fraction 0.9. The consensus contig from the mapping was obtained and inspected for ambiguities, which were corrected with reference to the original assembly or mapping. The open reading frame (https://www.expasy.org/) and annotation of the final sequences was carried out.

### Phylogenetic analysis

Nucleotide (nt) sequences of all 19 MDMV isolates obtained in this study and nine (including our previously MDMV-Bixby1 isolate)^25^ complete genome sequences (Table 1) available in the nucleotide sequence repository, GenBank were aligned with the ClustalW.v2 program^41^. Initially, neighbor joining (NJ) trees were developed for both complete genomes and individual genes. Then, based on the best model selection obtained with MEGA6, a Maximum Likelihood (ML) tree was constructed using the Tamura Nei model with 1000 bootstrap replicates for complete open reading frame (ORF) nt sequences. Individual gene trees were also developed using MEGA6 according to the best fitting model for each individual gene. For all phylogenetic trees, Sorghum mosaic virus (SrMV, accession no. U57358) was used as an out-group.

### Population genetic parameters

To understand the selection pressure on each coding region and complete ORF of MDMV isolates, the ratio of nonsynonymous (dN) to synonymous (dS) substitutions was calculated using DnaSP 5.10^42^. Site-specific selection pressure was measured for each codon site using different codon-based maximum-likelihood methods, SLAC, FEL, and MEME for positive selection with *p* = 0.1 available in Datamonkey 2.0 online version www.datamonkey.org^43^.

Nucleotide diversity (π) was calculated as average number of nucleotide differences per site between two sequences^44^.

### Neutrality tests

Neutrality tests such as Tajima’s D^45^, and Fu and Li’s D^46^ were also performed in DnaSP5.10 to understand the departure from neutrality for all mutations between group specific MDMV polyprotein sequences.

### Genetic differentiation and gene flow

The degree of genetic differentiation and gene flow among different populations of MDMV was measured in DnaSP5.10 using fixation index statistics (FST) as described in Ref.^47^.

### Recombination analysis

Recombination analysis were performed by RDPv4.69^48^, including seven different recombination detection algorithms (RDP, GENECONV, Chimaera, MaxChi, Bootscane, SISCAN and 3Seq). The complete genome sequences of all 19 MDMV isolates and 9 other complete genomes from GenBank (Table 1) were aligned and used in the RDP program. The recombination events considered acceptable were those detected by at least five or more recombination detection algorithms and were statistically significant with a Bonferroni-correct *P* value cutoff of 0.05.

### Ethics statements

Studies involving human and animals subjects. No animals or human studies are presented in this manuscript.

### Samples collection

All plant samples collected within the states were according to guidelines provided in an approved standard permit P526P-18-04623 issued by USDA-PPQ.

## Supplementary Information

Supplementary Figure S1.

Supplementary Table S1.

## Data Availability

The 19 complete genome sequences of MDMV isolates from the US presented in this study were deposited to NCBI database. The accession numbers from MW026050-MW026068 can be found online https://www.ncbi.nlm.nih.gov/genbank/.
