# Supplementary figures and images for: Evolutionary study of maize dwarf mosaic virus using nearly complete genome sequences acquired by next-generation sequencing
Source: Sci Rep. 2021 Sep 22;11:18786. doi: 10.1038/s41598-021-98299-9 (PMC8458484; doi:10.1038/s41598-021-98299-9)

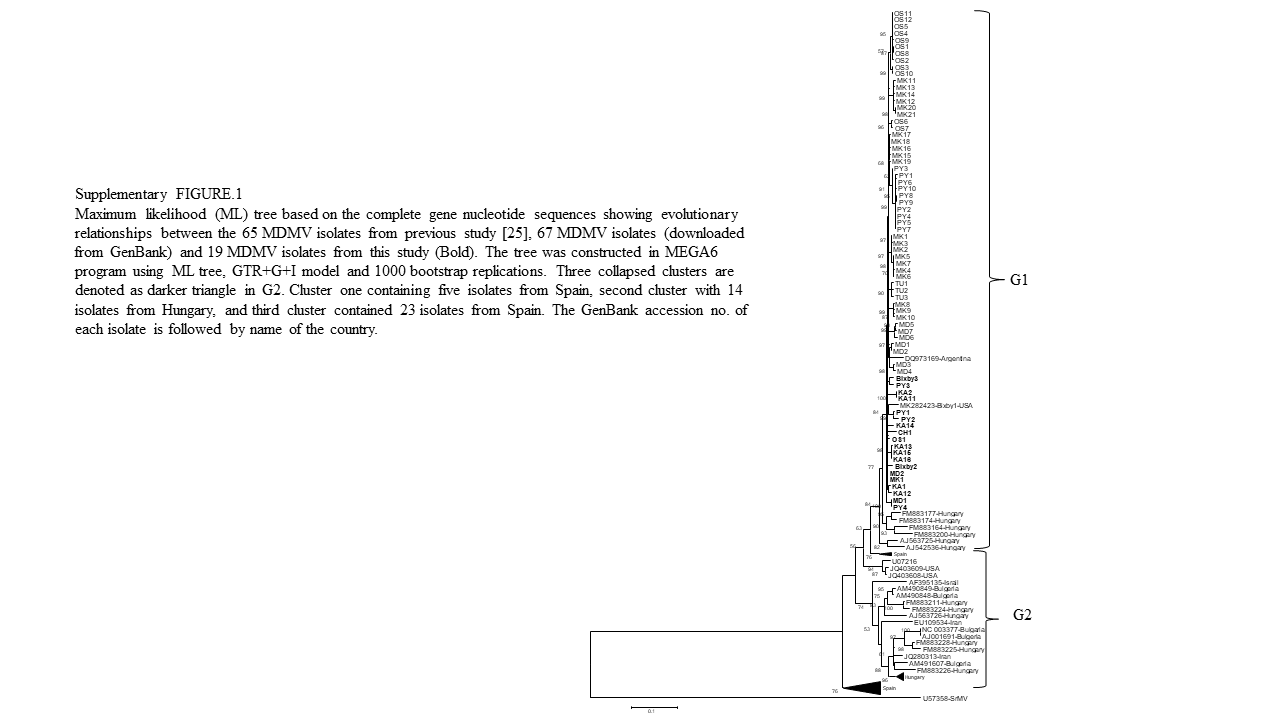

Supplement: Supplementary file 1 — Supplementary Figure S1. [file 41598_2021_98299_MOESM1_ESM.tif]
