# Supplementary material for: Evolutionary study of maize dwarf mosaic virus using nearly complete genome sequences acquired by next-generation sequencing
Source: Sci Rep. 2021 Sep 22;11:18786. doi: 10.1038/s41598-021-98299-9 (PMC8458484; doi:10.1038/s41598-021-98299-9)
Supplement: Supplementary file 2 — Supplementary Table S1. [file 41598_2021_98299_MOESM2_ESM.docx]

**Supplementary Table 1.** Complete genome sequences of 19 MDMV isolates from maize and Johnsongrass obtained by Illumina sequencing.

| Name of isolates | No. of reads | Reads for MDMV | Average length of reads | Total bases | Virus | % Coverage | Genome length (nt) | % Sequence identity^a^ |
| --- | --- | --- | --- | --- | --- | --- | --- | --- |
| Bixby2 | 451,352 | 27,818 | 144.13 | 65,051,977 | MDMV | 6% | 9225 | 95% |
| Bixby3 | 747,160 | 46,849 | 144.6 | 108,040,969 | MDMV | 6% | 9497 | 95% |
| CH1 | 847,802 | 239,592 | 144.26 | 122,301,431 | MDMV | 28% | 9453 | 95% |
| KA1 | 1,231,174 | 146,286 | 143.54 | 176,722,343 | MDMV | 34% | 9454 | 95% |
| KA2 | 262,548 | 94,086 | 147.8 | 38,616,697 | MDMV | 35% | 9391 | 95% |
| KA11 | 259,656 | 11,065 | 147.38 | 38,267,076 | MDMV | 4% | 9391 | 95% |
| KA12 | 542,514 | 199,393 | 147.34 | 65,051,977 | MDMV | 37% | 9431 | 95% |
| KA13 | 117,036 | 40,669 | 147.69 | 17,285,026 | MDMV | 23% | 9395 | 95% |
| KA14 | 260,882 | 93,736 | 147.04 | 36,360,917 | MDMV | 36% | 9440 | 95% |
| KA15 | 160,690 | 41,322 | 146.19 | 23,490,807 | MDMV | 26% | 9393 | 94% |
| KA16 | 235,510 | 84,099 | 147.57 | 34,353,704 | MDMV | 36% | 9439 | 95% |
| MK1 | 659,332 | 124,681 | 143.71 | 94,751,173 | MDMV | 19% | 9394 | 96% |
| OS1 | 550,774 | 105,578 | 144.04 | 79,335,548 | MDMV | 19% | 9415 | 95% |
| PY1 | 320,448 | 22,610 | 144.52 | 46,309,974 | MDMV | 7% | 9413 | 94% |
| PY2 | 1,246,282 | 367,965 | 144.36 | 179,941,920 | MDMV | 30% | 9204 | 94% |
| PY3 | 1,121,766 | 302,065 | 144.03 | 161,563,889 | MDMV | 27% | 9430 | 95% |
| PY4 | 597,388 | 166,000 | 144.43 | 86,283,638 | MDMV | 28% | 9444 | 95% |
| MD1 | 1,303,612 | 297,551 | 144.31 | 188,119,485 | MDMV | 23% | 9450 | 95% |
| MD2 | 196,152 | 69,047 | 144.42 | 28,328,952 | MDMV | 35% | 9491 | 95% |

^a^ Percent sequence identity compared to MDMV Bixby-1 isolate (MK282423) based on the complete genome nucleotide sequences of each isolate when blasted in the NCBI database.
